# Bacterial distribution in the Equatorial Indian Ocean using Amplicon sequencing of V3-V4 rDNA hypervariable region data

**DOI:** 10.1016/j.dib.2022.108673

**Published:** 2022-10-14

**Authors:** Alok K. Sinha, Bhaskar V. Parli, N. Anilkumar

**Affiliations:** a Ocean Science Group, National Centre for Polar and Ocean Research, Ministry of Earth Sciences, Headland Sada, Goa,403804; b O’ Smart, Hydrothermal Vent Group, National Centre for Polar and Ocean Research, Ministry of Earth Sciences, Headland Sada, Goa,403804

**Keywords:** Metagenomics, RAMA, Bacterial diversity, EIO

## Abstract

The Equatorial Indian Ocean (EIO) is a complex system strongly influenced by Indian Monsoon. During a RAMA (Research Moored Array for African-Asian-Australian Monsoon Analysis and Prediction) mooring maintenance expedition during the Southwest monsoon (August-September 2016) onboard *ORV Sagar Kanya,* seawater samples from the surface, deep chlorophyll maxima (DCM) and 200m were collected for bacterioplankton community structure. Herein we document our amplicon data of the bacterial community at 4 stations (4.01°S, 1.60°S, 0.36°N and 1.78°N) along the 67°00’ E transect. The samples were subjected to next-generation sequencing (NGS), followed by processing with Mothur v 1.48.0, and the taxonomic classification prepared with Silva 138.1nr reference database. Our data indicates *Alphaproteobacteria* (48 %) and *Cyanobacteria* (33 %) dominance in the surface and DCM samples.

**Specifications Table**

**Table:** 

Subject	Environmental Science
Specific subject area	Microbiology
Type of data	Table Graph Figure
How the data were acquired	Samples were collected using Niskin bottle (10 L sampler) (Seabird Inc., USA) attached to CTD rosette equipped with Sea bird CTD system (SBE911 plus, Sea-Bird Electronics, USA). Five litres (5 L) of water samples were filtered through 0.22-µm pore size, 47 mm diameter polycarbonate filters (Merck Millipore, USA). DNA was isolated using Power Water DNA kit (MoBio; USA) and sequencing was carried out using primer 341 F: 5’ CCTACGGGAGGCAGCAG 3’ and 806 R: 5′ GGACTACHVGGGTTCTAAT 3’ (Morris et al., 2018) with Hiseq Rapid V2 Kit.
Data format	Raw (All data were submitted in Fastq (Zip file) format)
Description of data collection	Amplicon data, hypervariable region (V3-V4) of bacterial 16S rDNA from Equatorial Indian Ocean
Data source location	Twelve water samples were collected from three different depths at four different stations during expeditions onboard ORV Sagar Kanya-333 (August- September 2016) (Latitude, Longitude, and depth provided in Table 1).
Data accessibility	The sequence has been submitted to the public repository Repository name: National Center for Biotechnology Information Data identification number: PRJNA876391 Direct URL to data: https://www.ncbi.nlm.nih.gov/bioproject/PRJNA876391/
Related research article	N/A

## Value of the Data

• Metagenomic data from the Equatorial Indian Ocean provides additional information on bacterial community structure in the relatively less explored oceanic region.
• Reported data will be used in the marine microbial diversity library construction
• Further metabolic prediction and applicational study will be quite easy with the availability of data from oligotrophic areas.
• Samples were collected from RAMA buoy sites, which monitor Indian monsoon activity, shall be useful in evaluating microbial response to changing Indian Ocean conditions.

## 1 Objective

The Equatorial Indian Ocean plays an important role in regional climate and global climate change through different processes like precipitation patterns in the surrounding land mass and shelter to a huge microbial diversity. To understand the monsoon pattern Ministry of Earth Science, India and NOAA conduct a collaborative objective of surface mooring deployment at different sites in the Indian Ocean. In order to understand the dynamic nature of the Indian Monsoon System, moorings are one of the important tools. Along with physical and chemical parameters, microbial samples were collected to understand bacterial diversity in specific locations. Collected microbial data shall aid in understanding the microbial distribution in the oligotrophic ocean like the Equatorial Indian Ocean in changing global climate.

## 2 Data Description

**Fig. 1:**
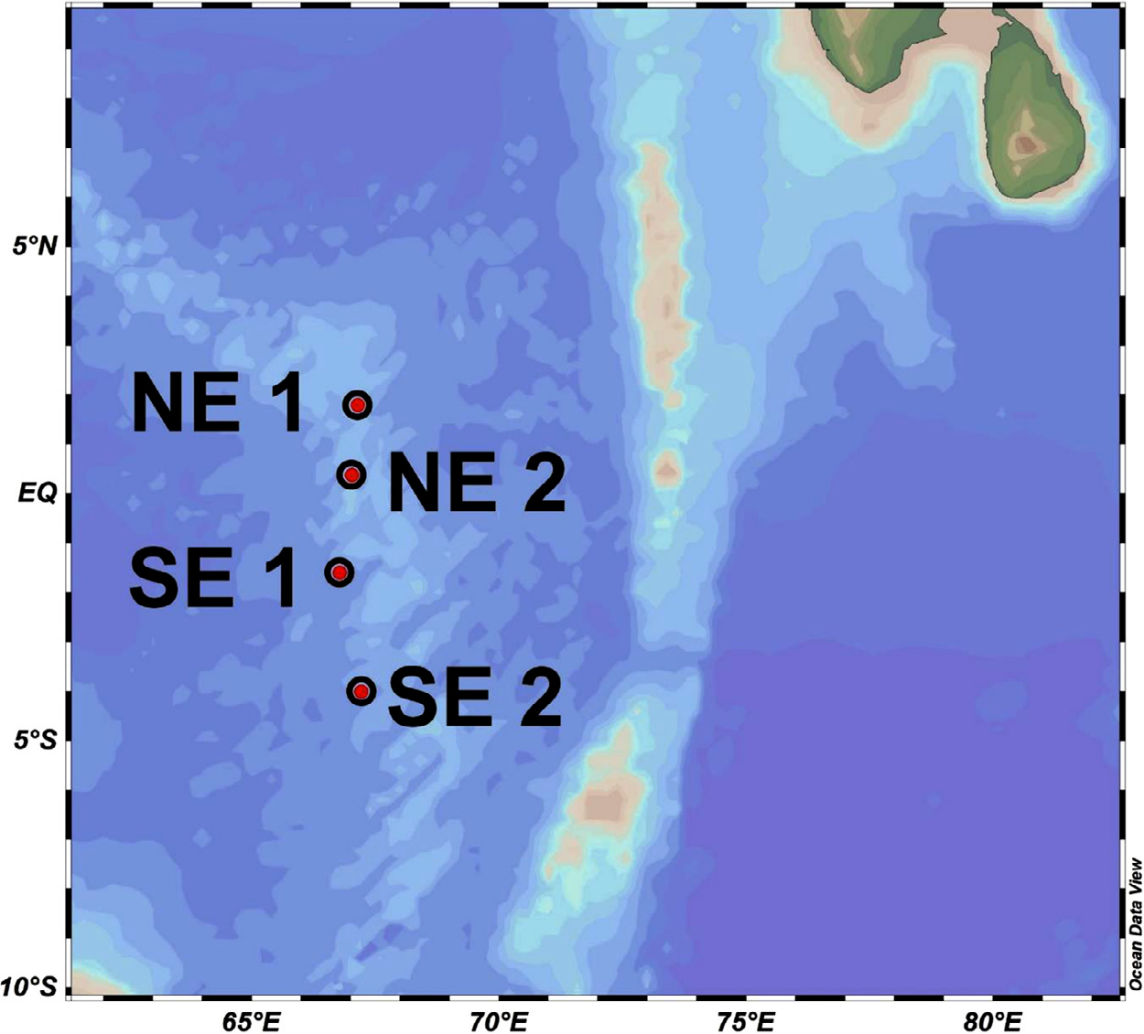
Map showing the sampling locations in Indian sector of Equatorial Ocean (EIO) during Sagar Kanya (SK) - 333 expedition (Geographical location in Table 1).

The sample was collected from four different locations in the Equatorial Indian Ocean (Fig. 1) at the RAMA (Research Moored Array for African-Asian-Australian Monsoon Analysis and Prediction) mooring site (Table 1). Hypervariable regions V3-V4 of 16s rDNA were sequenced using HiSeq sequencing techniques followed by bioinformatics processing using Mothur v 1.48.0 [1]. Taxonomic classification was carried out using Silva 138.1 nr database and further downstream processing of data was generated using MicrobiomeAnalyst [2].

**Table 1:** Detail of sequence summary of all the samples after sequencing and quality filtration.

Sample No	Sample ID	Latitude	Longitude	Depth	Total Read	GC contents	Phred Score
1	SE2_0m	-4.01	67.21	0 m	566924	52.14	37.73
2	SE2_DCM	-4.01	67.21	DCM	229411	51.32	37.24
3	SE2_200m	-4.01	67.21	200 m	627868	53.44	38.07
4	SE1_0m	-1.60	66.78	0 m	951887	51.31	36.67
5	SE1_DCM	-1.60	66.78	DCM	165689	51.77	36.94
6	SE1_200m	-1.60	66.78	200 m	536747	51.34	36.08
7	NE2_0m	0.36	67.03	0 m	542875	52.41	38.03
8	NE2_DCM	0.36	67.03	DCM	727186	50.35	36.21
9	NE2_200m	0.36	67.03	200 m	559834	52.91	37.81
10	NE1_0m	1.78	67.15	0 m	669312	52.23	37.85
11	NE1_DCM	1.78	67.15	DCM	137554	51.68	36.79
12	NE1_200m	1.78	67.15	200 m	1129375	51.92	35.51

**Fig. 2:**
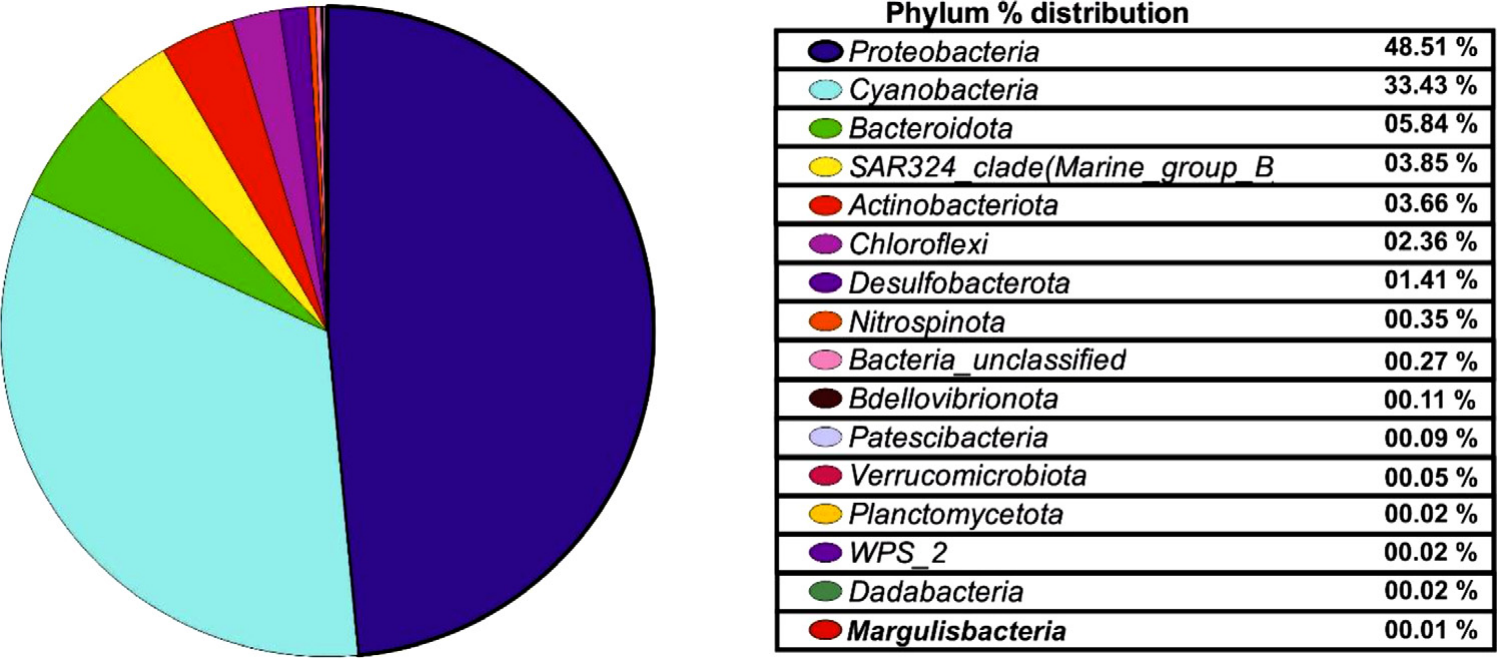
Pai Chart represent Percentage abundance (%) of different bacterial Phylum from accumulative processed data.

All phyla were excluded or merged with a count smaller than 10 taxa. After processing the taxonomic data, 13 different phyla were identified, with *Proteobacteria* (48.51%)*, Cyanobacteria* (33.43)*, Bacterioidata* (5.84%)*, SAR324 Clade marine group_B* (3.85%)*, Actinobacteriota* (3.66%)*, Chloroflexi* (2.36%)*,* and *Desulfobacteria* (1.41%) forming the major phyla while *Nitrospinota, Bdellovibrionota, Patescibacteria, Verrucomicrobiota, Planctomycetota, WPS_2, Dadabacteria,* and *Margulisbacteria* cumulatively constituted only 1% (Fig. 2)*.*

**Fig. 3:**
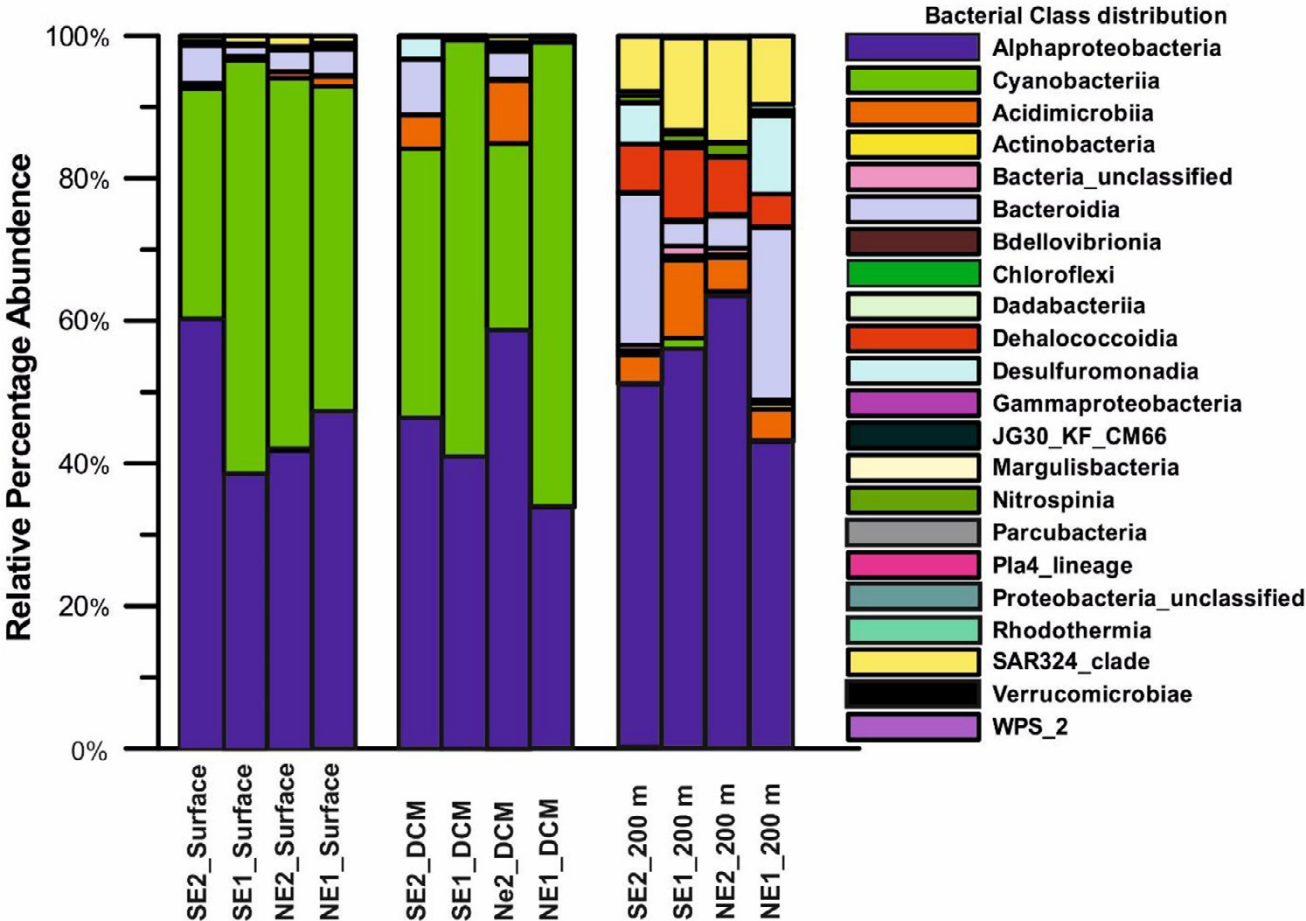
Relative abundance (%) of different bacterial class from sampling depths in Surface, DCM, and 200 m.

At Class-level distribution, *Alphaproteobacteria* (48 %) and *Cyanobacteria* (33 %) were the dominant classes in EIO waters covering 81% of the total microbial distribution. The rest of 19% of the microbial community was composed of 20 different classes of bacteria including *Gammaproteobacteria, SAR 324* etc (Fig. 3). At 200 m depth, spatial distribution of bacteria was more diverse compared to surface and deep chlorophyll maxima (DCM).

## 3 Experimental Design, Materials and Methods

Twelve water samples were collected from three different depths at four different stations (Table 1) during an expedition onboard ORV Sagar Kanya, cruise number 333 (August- September 2016). Aliquots of samples were collected using Niskin bottle (10 L sampler) (Seabird Inc., USA) attached to CTD rosette equipped with Sea bird CTD system (SBE911 plus, Sea-Bird Electronics, USA) [3]. Five litres (5 L) of water samples were filtered through 0.22-µm pore size, 47 mm diameter polycarbonate filters (Merck Millipore, USA) for bacterial diversity analysis. The filters were sealed in sterile tubes and stored at -80°C and transported to the laboratory for DNA extraction [4].

### 3.1 DNA Isolation and Sequencing

Total DNA was extracted from 0.22 μm pore-size polycarbonate membrane filter using Power Water DNA kit (MoBio; USA). The hypervariable region (V3-V4) of bacterial 16S rDNA was amplified using primer 341 F: 5’ CCTACGGGAGGCAGCAG 3’ and 806 R: 5′ GGACTACHVGGGTTCTAAT 3’ [5] with Hiseq Rapid V2 Kit for 2*250 base pair (bp) sequence. DNA sequencing was outsourced to M/s Agrigenome Laboratory Ltd. (India). The raw data of the V3-V4 sequence were deposited NCBI database. Quality scores and CG base were checked and processed for down streaming bioinformatics analysis (Table 1).

### 3.2 Bioinformatics and Statistics Analysis

Downstream processing of DNA sequences was carried out using Mothur V-1.48.0 (Log file attached as supplementary 1). Contigs were prepared for total 6844662 reads. These contigs were trimmed and around 3852596 sequences were removed using screen.seqs command. Thereafter, 2992066 sequences were selected and thereafter 1624092 sequences have been selected as unique sequences. These selected unique sequences were further aligned using Silva.nr_v138.1 database. Further, sequential downstream processing was carried out and a total 1924212 sequences were obtained and subsampled with reference to the smallest group of 32518 sequences. Chloroplast, mitochondria, Eukaryota, Archaea and unknown samples were removed using remove.lineage command and further shared and taxonomy files were created with cutoff value of 0.03. Grapher 10, Microbiomeanalyst and R softwares were used for downstream processing and final data preparation.

## Ethics Statements

This article does not contain any studies with human participation or animal performed by any of the authors.

## CRediT Author Statement

**Alok K. Sinha:** Data curation, Methodology, Formal analysis, Writing – original draft; **Bhaskar V. Parli:** Visualization, Investigation, Writing – review & editing, Supervision, Funding acquisition; **N. Anilkumar:** Project administration, Funding acquisition, Resources.

## Declaration of Competing Interest

The authors declare that they have no known competing financial interests or personal relationships that could have appeared to influence the work reported in this paper.

## Data Availability

Southern Ocean Carbon Processes (Original data) (NCBI).

## Appendix Supplementary materials

Image, application 1

## References

[1] J.J. Kozich, S.L. Westcott, N.T. Baxter, S.K. Highlander, P.D. Schloss, Development of a Dual-Index Sequencing Strategy and Curation Pipeline for Analyzing Amplicon Sequence Data on the MiSeq Illumina Sequencing Platform, (2013). 10.1128/AEM.01043-13. PMC3753973 23793624

[2] Dhariwal A., Chong J., Habib S., King I.L., Agellon L.B., Xia J. (2017). MicrobiomeAnalyst: a web-based tool for comprehensive statistical, visual and meta-analysis of microbiome data. Nucleic Acids Res..

[3] Sinha Alok K., Bhaskar P.V., Tripathy S.C., Sarkar A., Prabhakaran S. (2019). Effects of growth conditions on siderophore producing bacteria and siderophore production from Indian Ocean sector of Southern Ocean. J. Basic Microbiol..

[4] Sadaiappan B., Kannan S., Palaniappan S., Manikkam R., Ramasamy B., Anilkumar N., Subramanian M. (2020). Metagenomic 16S rDNA amplicon data of microbial diversity and its predicted metabolic functions in the Southern Ocean (Antarctic). Data Brief.

[5] Morris L., O'Brien A., Natera S.H.A., Lutz A., Roessner U., Long S.M. (2018). Structural and functional measures of marine microbial communities: An experiment to assess implications for oil spill management. Mar. Pollut. Bull..

